# Growth, infection, and humoral immunity in children who are HIV exposed and uninfected

**DOI:** 10.3389/fcimb.2026.1816838

**Published:** 2026-07-09

**Authors:** Romeo Djounda, Modeste R. Ngamaleu, Awanakam Honore, Moritz Schmiedeberg, Kevine Tchamda, Martial Tsague, Eva Gutenkunst, Jude Bigoga, Rose Leke, Charles Kouanfack, Micheal Besong, Krystelle Nganou-Makamdop, Forgu Esemu Livo

**Affiliations:** 1 Immunology Laboratory, The Biotechnology Center, University of Yaoundé I, Yaoundé, Cameroon; 2 Laboratory of Fundamental Virology, Centre for Research on Emerging and Reemerging Diseases, Institute of Medical Research and Medicinal Plant Studies, Ministry of Scientific Research and Innovation, Yaoundé, Cameroon; 3 Department of Biochemistry, University of Yaoundé I, Yaoundé, Cameroon; 4 Department of Immunology, Catholic University of Central Africa, Yaoundé, Cameroon; 5 Harald zur Hausen Institute of Virology, Uniklinikum Erlangen, Erlangen, Germany; 6 Department of Medicine 3, Uniklinikum Erlangen, Erlangen, Germany; 7 Department of Biomedical Sciences, Faculty of Health Sciences, University of Buea, Buea, Cameroon

**Keywords:** growth, HEU, humoral immunity, infection, sex

## Abstract

**Background:**

Children who are HIV-exposed uninfected (HEU) show greater morbidity and mortality than HIV-unexposed children (HUU). In this study we investigate sex differences in growth, infection rates and antibody response among HEU and HUU infants.

**Methods:**

The study enrolled 107 pregnant women with HIV and 103 pregnant women without HIV with follow-up of their infants from birth to 12 months of age. Study measures assessed included growth parameters, the prevalence of children with overt disease symptoms as reported by the mother, PCR-based assessment of infections (cytomegalovirus (CMV), respiratory syncytial virus (RSV), rhinovirus, influenza A & B, rotavirus and malaria) as well as antibody profile to CMV, RSV and enterovirus infections.

**Results:**

Compared to male HUU, male HEU infants had higher stunting at month 3 (0% vs 17.4%; P = 0.01) and month 12 (12.5% vs 38.9%; P = 0.03). Additionally, they showed transiently lower Weight-for-age-z-scores at 3 months (1.07 vs 0.05, P = 0.04), with higher risk of rhinorrhea (RR = 2.29, P = 0.02) and lower enterovirus titers at birth (P = 0.0066). Female HEU showed transiently higher stunting at 6 months (0% vs 21%; P = 0.01) and lower CMV viremia at 6 months, with elevated CMV antibody titers at 3 months (P = 0.04) compared to female HUU. With prevalence ranging from 25%–61%, CMV and Rhinovirus infections were dominant in all groups. HEU and HUU exhibited similar antibody decay and acquisition patterns for CMV, RSV, and Enterovirus.

**Conclusion:**

Overall, HEU and HUU infants showed similar growth, infection and humoral immune profiles. However, exploratory analyses identified transient sex-specific differences, with male HEU infants displaying poorer growth trajectories and selected infection-related vulnerabilities, highlighting the need for larger studies to clarify sex-dependent effects of *in utero* exposure to HIV/ART.

## Background

The implementation of the prevention of mother to child transmission (PMTCT) has greatly averted the number of perinatal HIV infections. This has led to a steady increase in the population of children who are HIV-exposed but uninfected (HEU) as born to cART-treated women living with HIV(WLHIV). Currently there are over 16 million HEU worldwide with over 90% of them living in Sub Saharan Africa where the burden of HIV remains highest (UNAIDS, 2023). Despite being uninfected with HIV, high mortality and morbidity rates have been reported amongst the HEU when opposed to their HIV unexposed uninfected (HUU) peers (Slogrove et al., 2016; Nlend et al., 2023; Lwanga et al., 2024) Compared to HUU, HEU have been shown to have greater infection rates, infection related hospitalization and prolonged hospitalization (Slogrove et al., 2012; Cohen et al., 2016; Lwanga et al., 2024; Wedderburn et al., 2024; Infectious disease morbidity and growth among young HIV-exposed uninfected children in Jamaica, 2025). Moreover, several studies report high prevalence of stunting and wasting amongst the HEU infants during their first 2 years of life (Sudfeld et al., 2016; Fowler et al., 2022; Tshiambara et al., 2023; Tiwari et al., 2025). The reasons for these differences are multifactorial and poorly understood. It has been hypothesized that exposure to cART *in-utero* and intrauterine chronic viral infections such as HIV antigens and CMV, reduced duration of breastfeeding, reduced maternal transfer of antibodies during gestation and breastfeeding, and reduced subsequent immune modulation in HEU infants are possible mechanisms that underpin differences in HEU and HUU infants (Salvi et al., 2025). The importance of humoral immunity is crucial since the infants rely on acquired maternal antibodies during their first months of life while their immune system fully develops (Semmes et al., 2021). Any quantitative and qualitative abnormalities in maternal antibody transfer and ulterior dynamics may cause imbalances in immune response against common infections. Infections with malaria, enteroviruses, cytomegalovirus (CMV) and respiratory syncytial virus (RSV) are major contributors to child morbidity and mortality among infants in low and middle-income countries (LMIC). Evidence shows that HEU infants experience reduced transfer of maternal antibodies to malaria, RSV and Influenza B (Impaired Haemophilus influenzae Type b Transplacental Antibody Transmission and Declining Antibody Avidity through the First Year of Life Represent Potential Vulnerabilities for HIV-Exposed but -Uninfected Infants | Clinical and Vaccine Immunology; Patel et al., 2019; Ray et al., 2019). The latter added to exposure to cART and HIV *in-utero*, might enhance susceptibility to infections. Despite the clinical importance of these infections, their true burden is unknown, and host response is understudied in HEU infants. Most studies have focused on individual aspects of HEU’s health, describing infection burden to single infections or evaluating immune responses in isolation. In addition, sex differences in infection dynamics and immune response are increasingly recognized and studied, but not extensively in HEU (Steeg and Klein, 2016). To address this gap, we recorded growth parameters, self-reported disease symptoms, infection burden for CMV, malaria, rotavirus, rhinovirus, RSV, Influenza A and B, and antibody dynamics to CMV, RSV and Enterovirus over the first year of life, and performed sex stratified analysis in a longitudinal cohort of HEU and HUU. These aspects put together can give a holistic view of overall trends in morbidity in HEU compared to their HUU counterparts.

## Methodology

### Study site and participants

This study was a prospective cohort study conducted in Yaoundé, Cameroon, at the CASS Nkoldongo hospital from June 2022 to May 2024. Pregnant women living with and without HIV were recruited in the third trimester of pregnancy and were received during delivery for the recruitment and follow-up of their HEU and HUU until 12 months of age. Inclusion in the study was restricted to women that had uncomplicated pregnancies and were either HIV infected on cART or uninfected and were not planning to relocate in another city in the next two years. Aligned with the national immunization schedule, study visits occurred at 6, 10 and 14 weeks (3 months) then at 6, 9 and 12 months.

This study was approved by the Cameroon Baptist Convention Health Boards’ Institutional Review Board (IRB2022-05) and all participants provided written informed consent. Research was conducted in accordance with the ethical standards of the Helsinki Declaration.

### Anthropometric and clinical assessments

Data collected during each visit included anthropometric measures (weight, length/height, mid upper arm circumference and head circumference) and clinical parameters (maternally-reported history of infection, hospitalization and symptoms). Major symptoms reported were solely based on mother observations: fever (perceived rise in temperature either by touch or using a thermometer: axillary temperature above ≥ 38 °C), cough (forceful exhalation of air, sometimes sounding dry, wet or backing), diarrhea (waterier and more frequent stool in 24H), rhinorrhea (drainage of liquid or mucus from the nasal passage), skin rash (appearance of red spots, patches or irritation on the skin) and vomiting (forceful spiting of stomach contents). Growth Z-scores; weight-for-age (WAZ) length/height for age (HAZ), head circumference-for-age (HCZ), body mass index for age (BAZ) and weight height-for-age (WHZ) were calculated according to World Health Organization (WHO) standards using the WHO Anthro survey Analyzer.

### Sample collection and processing

Venous blood samples were collected from mothers at recruitment and from infants at birth (cord), 14 weeks, 6, 9 and 12 months. Maternal and cord plasma was separated and stored at -80 °C. At all infants timepoints, aliquots of 100ul whole blood were stored in 100ul DNA/RNA shield (DNA/RNA shield™, ZYMO RESEARCH) and stored at -20 °C. Infants buccal, Nasal and Rectal swabs were also collected at 14 weeks, 6, 9 and 12 months and were conserved in DNA/RNA shield diluted 1:1 and frozen at -20 °C.

### Detection, of pathogen infections by RTPCR

Nucleic acids were extracted from buccal, nasal, rectal swabs and whole blood for the screening of CMV, RSV, Rhino virus, Influenza A and B (Infl A & B), Rota virus, and malaria using ZYMO_RESEARCH extraction kits following manufacturers procedures. To screen for CMV, DNA was extracted from buccal swabs using Quick-DNA/RNA™ viral kit. Likewise, to screen for malaria, DNA was extracted from whole blood specimens using Quick-DNA™ miniprep kit. To screen for RNA viruses (RSV, Rhinoviruses, Infl A & B and Rota viruses), RNA was extracted using the Quick-RNA™ viral kit. Amplification was conducted by quantitative real-time PCR (qPCR) for DNA and reverse transcription qPCR (RT-qPCR) for RNA on the Bio-Rad CFX_Opus_96 Real-Time PCR platform. Plasmid standards were also used to generate ten-fold serial dilutions (10^1–^10^7^ copies/µL) for absolute quantification. CMV was co-amplified with Albumin, to allow for normalization of cell input. All reactions were run with specific primers and probes using Luna Universal Probe One-Step RT-qPCR Kit w/o ROX and Luna Universal Probe qPCR Master Mix **Supplementary Table 1** **and** **2**. Viral loads below the lower limit of detection (10copies/µL) were arbitrarily attributed a viremia of 8 copies/µL. CMV viremia was normalized to that of Albumin (two copies/cell) and expressed as per 10^5^ cells using the ratio of CMV to albumin copy numbers.

### Antibody measurements

CMV, RSV and Enterovirus specific total IgG antibodies were measured by semi-quantitative ELISA with luminescence readout for broad dynamic range. High binding ELISA plates were coated with recombinant CMV gB, RSV cell culture extract and recombinant enterovirus antigen (from Serion_Immunologics) at concentration of 1µg/ml in carbonate buffer (15mM NaCO3, 35mM NaHCO3 in H2O pH 9.6) and stored overnight at RT. Plates were blocked with 5% skimmed milk in PBS-T20 (PBS and 0.05% Tween-20) for 2h at RT. Plasma samples were diluted 1:200 in 2% skimmed milk PBS-T20 and incubated for 2h at RT. Goat anti-human IgG-HRP (1:3000) was then added and incubated for 1h at RT. After every incubation step, the plate was washed 3 times with PBS-T20. Subsequently, plates were washed twice with PBS prior to adding ECL solution and immediate measurement of relative light units per second (RLU/s) using a microplate luminometer (Molecular Devices).

### Statistical analysis

Data collected was analyzed using GraphPad prism 10.6.0. Gaussian distribution of data sets was tested and corresponding parametric and non-parametric tests were applied for the comparison of continuous data. Paired tests were used to assess differences within dependent datasets. For analysis of categorical data, fishers exact or chi square tests were used. All analysis was based on available data. To assess the independent effect of HIV exposure on growth parameter over time, mixed effect model regression analysis was fitted using STATA 15.0. Covariates included Socioeconomic Status (SES), maternal and gestational age, feeding mode. All available repeated measurements were included and no imputation was performed assuming data was missing at random. For model parsimony, covariates that did not influence growth trajectories were excluded from model (maternal age and gestational age). Correlation analysis of antibody titers was performed using R software (version 4.5.1). P<0.05 defined significance.

## Results

### Participant characteristics

**Figure 1:**
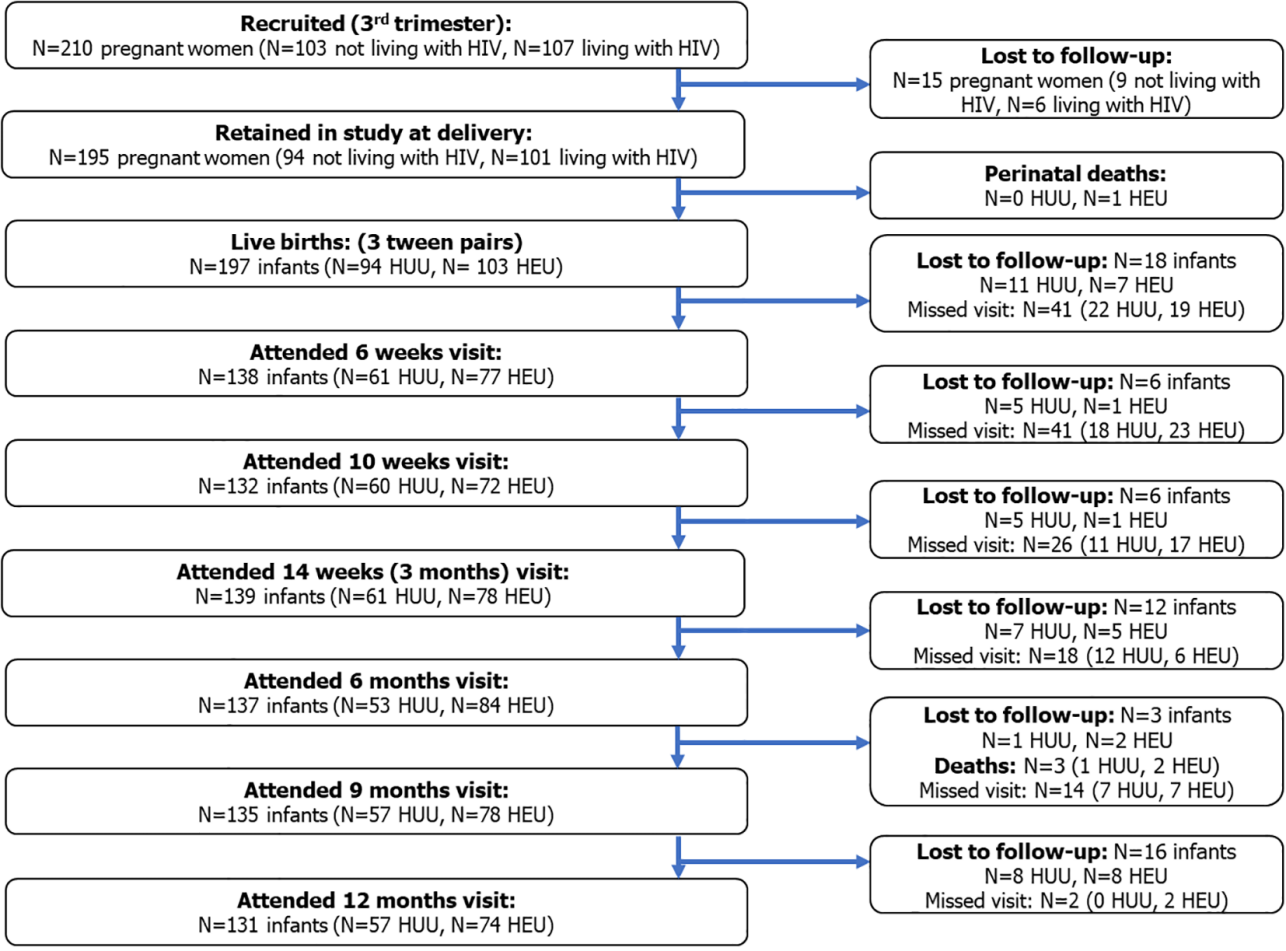
Flow diagram of included participants. Number of participants included in the study from recruitment of pregnant women through follow-up during the first 12 months of life. HEU, children who are HIV- exposed and uninfected; HUU, children who are HIV-unexposed and uninfected; HIV, human immunodeficiency virus.

A total of 210 pregnant women were recruited among which 107 were living with HIV on cART and 103 without HIV. Of these, 195 were retained at delivery with 196 live births (94 HUU and 103 HEU) after excluding perinatal deaths. After excluding postnatal deaths, 193 children participated in at least one of the 6 scheduled visits with a retention rate of 63.8% with no perinatal HIV recorded at 6 weeks routine HIV-1 PCR (**Figure 1**).

When compared to pregnant Women living without HIV, women living with HIV were older (32 SD ± 5.7 vs 28 SD ± 5.4: P<0.0001) and multigravida. Women living with HIV were all on ART with most of them (76/107 [71%]) receiving the standard first-line ART regimen consisting of tenofovir_disoproxil_fumarate (TDF), lamivudine (3TC), and efavirenz (EFV) while 31/107 (29%) were on other combinations. The median duration on ART was 70 months (IQR = 23.3 – 103.8) **Table 1**.

**Table 1:** Maternal and child baseline characteristics.

Parameter	Overall (N = 210)	Women not living with HIV or HUU (N = 103)	Women living with HIV or HEU (N = 107)	P value
Sociodemographic parameters				
Age in years, mean (SD)^b^	30 (± 5.7)	28 (± 5.4)	32 (± 5.7)	<0.0001
**Weight (kg), median (IQR)** ^a^	76 (68.3–86.5)	76 (69–86)	76 (68–86)	0.39
Level of education, N (%)^c^				
None	1 (0.5)	0 (0)	1 (1.0)	0.98
Primary	28 (13.3)	14 (13.6)	14 (13.1)	0.91
Secondary	136 (64.8)	66 (64.1)	70 (65.4)	0.84
University	45 (21.4)	23 (22.3)	22 (20.7)	0.76
Marital status, N (%)^c^				
Single	53 (25.2)	20 (19.4)	33 (30.8)	0.71
Married	32 (15.2)	15 (14.6)	17 (15.9)	0.79
Cohabitation	124 (59.0)	68 (66.0)	56 (52.3)	0.04
Divorced	1 (0.5)	0 (0.0)	1 (1.0)	0.98
**Profession (Employed), N (%)** ^c^	95 (45.2)	45 (43.7)	50 (46.7)	0.66
**Monthly income (FCFA), median (IQR)** ^a^	100K (70K–110K)	100K (60K–100K)	100K (71.3K–120K)	0.27
Clinical parameters				
**HBV positive, N (%)** ^c^	7 (3.3)	3 (2.9)	4 (3.7)	0.74
**Hemoglobin (g/dL), mean (SD)**	11.10	11.3 (± 1.3)	10.9 (± 1.1)	0.04
**Multigravida, N (%)** ^c^	176 (83.8)	78 (75.7)	98 (91.6)	0.002
Parity, N (%)^c^				
Nullipara	35 (16.7)	23 (22.3)	12 (11.2)	0.04
Primipara	36 (17.1)	16 (15.5)	20 (18.7)	0.59
Multipara	137 (65.2)	62 (60.2)	75 (70.1)	0.15
Missing	2 (1.0)	2 (1.9)	0 (0)	
ART regimen, N (%)				
TDF/3TC/DTG	/	NA	6 (5.6)	
TDF/3TC/EFV	/	NA	76 (71.0)	
Other	/	NA	2 (1.9)	
Missing	/	NA	23 (21.5)	
**Duration on ART (months), median (IQR)** ^a^	/	NA	70 (23.3–103.8)	
**Cotrimoxazole prophylaxis, N (%)**	/	NA	44 (41.0)	
Birth parameters				
**Infant sex N (%)** ^c^				0.78
Female	113 (53.1)	56 (54.3)	57 (51.8)	
Male	100 (46.9)	47 (45.7)	53 (48.2)	
Term of delivery, N (%)^c^				
Preterm (<37 weeks)	0 (0)	0 (0)	0 (0)	/
Term (37–42)	198 (93.0)	94 (91.3)	104 (94.6)	0.43
Post-term (>42)	0 (0)	0 (0)	0 (0)	/
Missing	15 (7.0)	9 (8.7)	6 (5.4)	
Mode of delivery, N (%)^c^				
Per vaginal	183 (85.9)	89 (86.4)	94 (85.5)	>0.99
C-section	10 (4.7)	3 (2.9)	7 (6.3)	0.34
Missing	20 (9.4)	11 (10.7)	9 (8.2)	
**Birth weight (g), median (IQR)** ^a^	3250 (3060-3515)	3295 (3100-3538)	3240 (3035-3515)	0.41
Normal (>2500 g), N (%)^c^	181 (85.0)	87 (84.5)	94 (85.5)	0.85
Low (1500–2500 g)	12 (5.6)	5 (4.8)	7 (6.3)	0.78
Missing	20 (9.4)	11 (10.7)	9 (8.2)	
**Birth length (cm), mean (SD)**	50.4 (± 2.6)	50.6 (± 2.5)	50.3 (± 2.8)	0.49
**Head circumference (cm), median (IQR)** ^a^	35.0, (34-36)	35.0, (34-36)	36.0, (34-36)	0.05
Infant feeding (first 6 months), N (%)^c^ (Percentages are based on the overall cohort during the first 6 months of life)/				
Exclusive breastfeeding	46 (21.6)	19 (18.4)	27 (24.5)	0.26
Formula feeding	36 (16.9)	2 (1.9)	34 (30.9)	<0.0001
Mixed feeding	53 (24.9)	31 (30.2)	22 (20.1)	0.11
Missing	78 (36.6)	51 (49.5)	27 (24.5)	

^a^ Mann-Whitney test;

^b^ Unpaired t test,

^c^ Chi Square test, K= × 1000.

Birth outcomes (term of delivery, mode of delivery, birth weight, birth length and head circumference) were similar between HUU (103) and HEU (110) **Table 1**. Most infants in both groups HUU (91.3%) and HEU (94.6%) were born at term by vaginal delivery (HUU = 86.4%, HEU = 85.5%) and had a normal weight (HUU 84.5% and HEU = 85.5%). Median birth weight was 3295g; IQR = 3100g-3538g and 3240; IQR = 3035g-3515g in both HUU and HEU groups respectively. During the first six months of life, the proportion of HUU and HEU infants who were exclusively breastfed were similar (18.4% and 24.5%; 0.26 for HUU and HEU respectively). This was also similar observed for mixed feeding (30.2% and 20.1%; P = 0.11 for HUU and HEU respectively). Nevertheless, the proportion of HEU on formula feeding was higher than HUU (1.9% and 30.9% for HUU and HEU respectively; P=<0.0001) with 61.7% (21/34) of the formula fed HEU being male infants **Table 1**. Compared to HUU, more HEU infants were not breastfed from 3 to 12 months **Supplementary Figure 1**.

### Growth outcomes in HEU vs HUU

**Figure 2:**
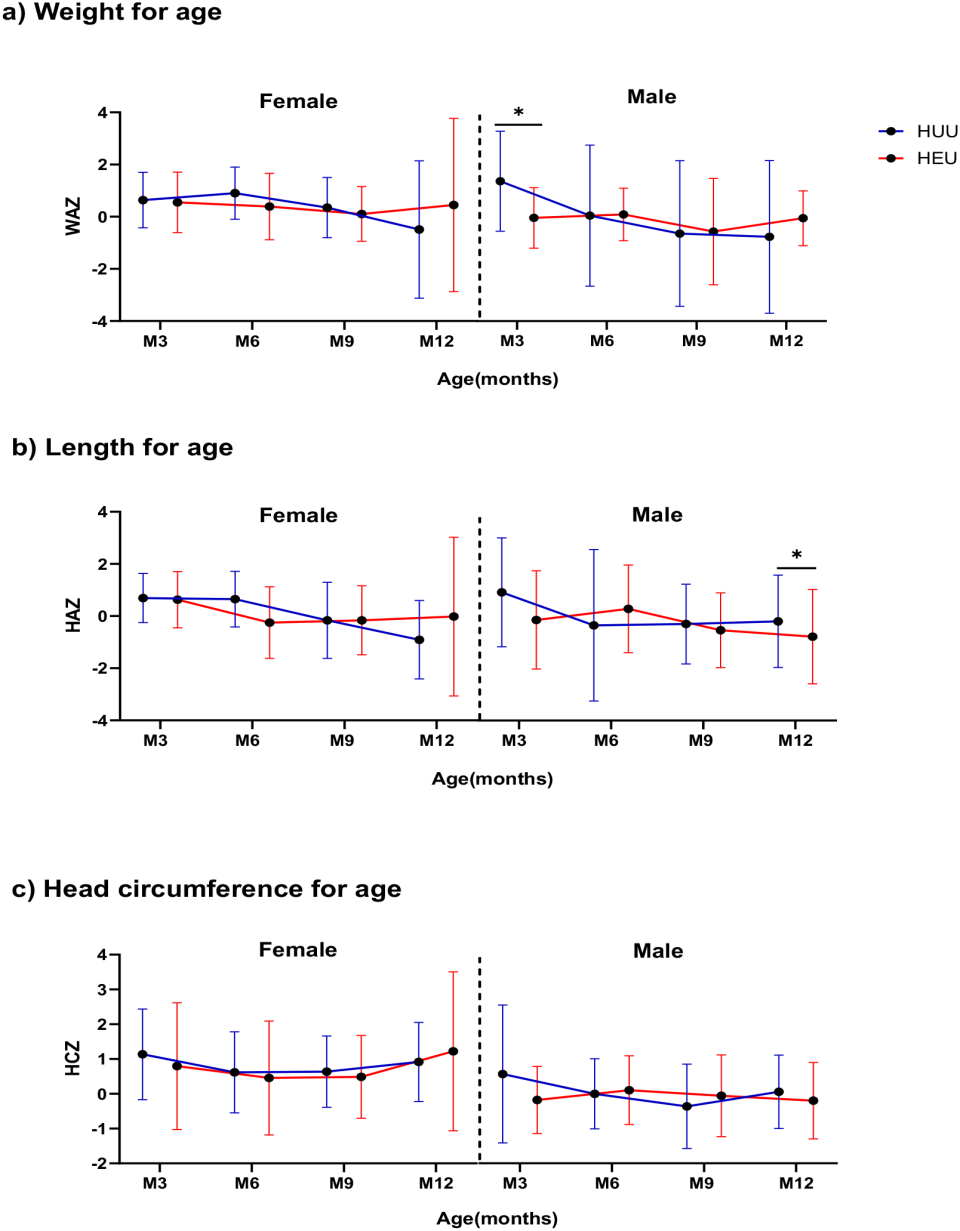
Growth curves for **(a)** Weight, **(b)** Length and **(c)** Head circumference for age measured at 3, 6, 9 and 12 months of age in female and male HUU and HEU infants plotted as means and standard deviation using z-scores. HUU: Female N = 18, Male N = 17. HEU: Female N = 30, Male N = 28. Note: The dependent t test was used for normally distributed data while the Wilcoxon matched-pairs signed rank test was used to compare non-normally distributed data sets.

Baseline sex stratified comparisons of HEU and HUU infants WHZ, HAZ, WAZ, BAZ and HCZ Z-scores across 4 timepoints (months 3, 6, 9, and 12) revealed at month 3, that male HEU experienced low WAZ (1.07 ± 1.94 vs 0.05 ± 1.35; P = 0.04 for HUU and HEU respectively) and low HCZ (1.39 ± 2.38 vs 0.43 ± 1.17; P = 0.03 for HUU and HEU respectively) compared to their HUU peers. No other sex-based differences were observed in HEU vs HUU **Supplementary Table 3**. When analysis was restricted to paired samples in the cohort, the difference in WAZ observed among males at month 3 persisted (P = 0.04) **Figure 2A**. Infants’ nutritional classification by WHO criteria were similar except for stunting which was more prevalent in female HEU at month 6 (0% vs 21%; P = 0.01) and among male HEU at month 3 (0% vs 17.4%; P = 0.01) and month 12 (12.5% vs 38.9%; P = 0.03) compared to their HUU counterparts’ **Supplementary Table 4**. In males the difference observed at month 6 persisted to month 12 (P = 0.04) **Figure 2B**.

To ascertain if these differences were driven by maternal HIV, mixed-effects models while accounting for repeated measures (3, 6, 9, and 12 months) were fitted. In unadjusted models, male HEU infants had lower HAZ compared with HUU infants (β –0.73; 95% CI –1.44, –0.02; P = 0.043), whereas no significant associations were observed for other growth outcomes in either sex **Supplementary Table 5**. Adjusting for socioeconomic status (SES), mode of feeding, maternal and gestational age, no significant associations persisted between HEU and HUU across all growth parameters both in females or males **Supplementary Table 5**. Similar observations were made after performing sensitivity analysis excluding formula fed infants although HCZ persisted among female HEU infants (β –1.10; 95% CI –2.11, –0.10; P = 0.031) **Supplementary Table 6**. Among HEU infants, maternal ART duration was not associated with any growth outcome in either females or males **Supplementary Table 7**. Overall, HEU exhibited transient sex-specific growth vulnerabilities which were attenuated after adjusting for multiple confounding factors.

Note: The dependent t test was used for normally distributed data while the Wilcoxon matched-pairs signed rank test was used to compare non-normally distributed data sets.

### Sex-stratified differences in symptom prevalence among HEU and HUU

**Figure 3:**
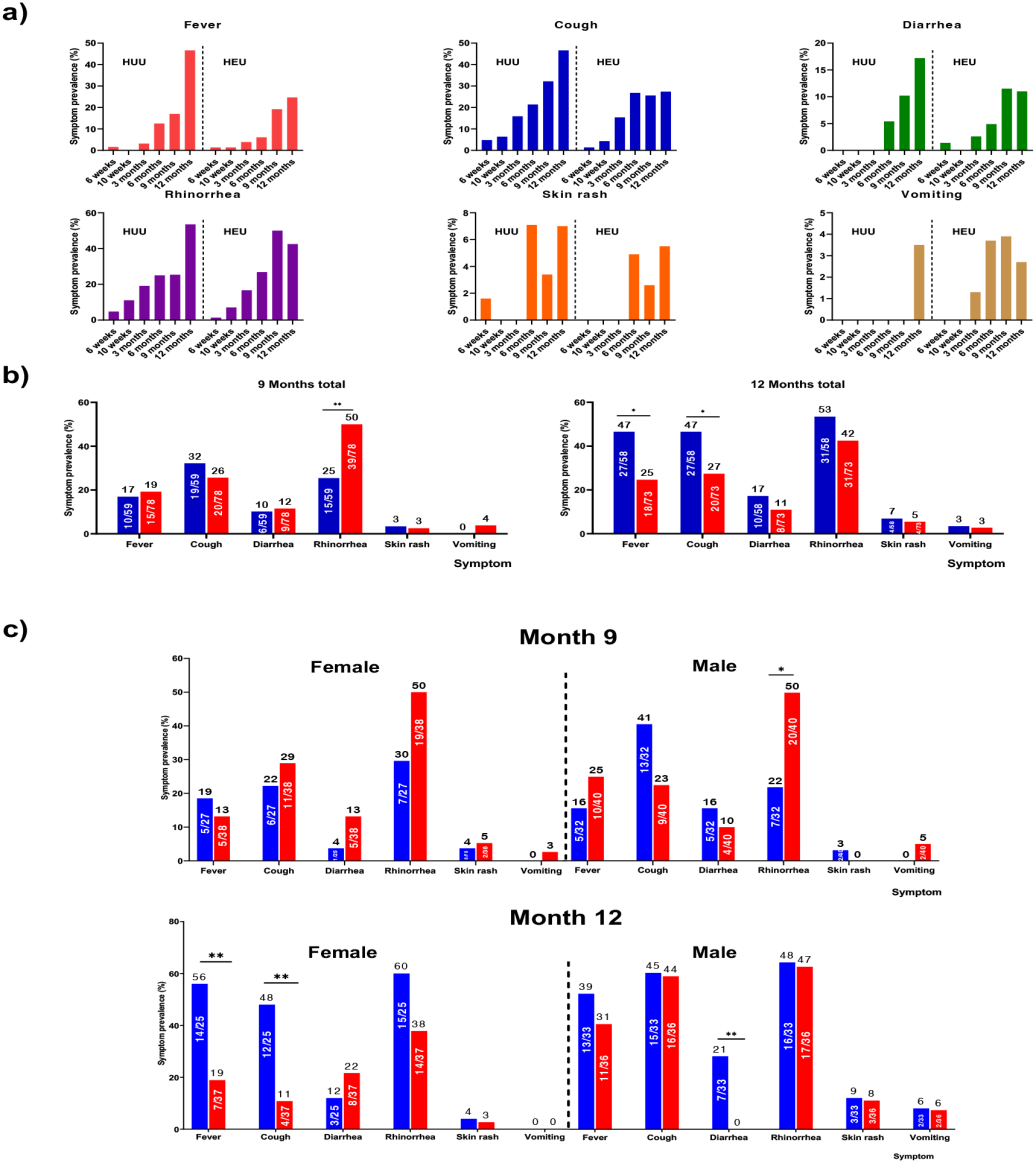
Longitudinal trend and prevalence of maternal self-reported Symptoms (fever, cough, diarrhea, rhinorrhea, skin rash, and vomiting) in HEU (red) and HUU (blue) Infants during the First Year of Life. **(a)** Increase in prevalence of symptoms from 6 weeks to 12 months. **(b)** HEU experience higher prevalence of rhinorrhea at month 9. **(c)** Male HEU experience higher rates of rhinorrhea at month 9. Note: *Pearson’s Chi-square/Fisher’s exact test was used for categorical data to determine the differences in symptom rates in HEU and HUU infants.*.

Irrespective of the groups, the prevalence of self-reported illness symptoms increased from 6 weeks to 12 months of age **Figure 3A**. HEU were twice more likely to have rhinorrhea-related symptom than HUU at 9 months of age (RR = 2.00; 95% CI 1.23, 3.26; P = 0.005). HEU had a 0.53 lower RR of acquiring a fever (95% CI 0.32, 0.85; P = 0.01) and a 0.59 RR of developing a cough (95% CI 0.37, 0.93; P = 0.03) than their HUU counterparts at month 12 **Figure 3B**. HEU males but not females had more rhinorrhea symptoms than HUU counterparts at month 9 (RR = 2.29; 95% CI 1.17, 4.79; P = 0.02) whereas at month 12 the HEU female but not male showed lower prevalence of reported fever (RR = 0.31; 95% CI 0.15, 0.63; P = 0.002) and cough (RR = 0.23; 0.23; 95% CI 0.08, 0.58; P = 0.002) compared to the female HUU **Figure 3C**. Overall, illness symptoms increase with age in both groups with male HEU displaying higher rhinorrhea at 9 months.

### Prevalence and viremia of infections in HEU and HUU stratified by HIV exposure and sex

**Figure 4:**
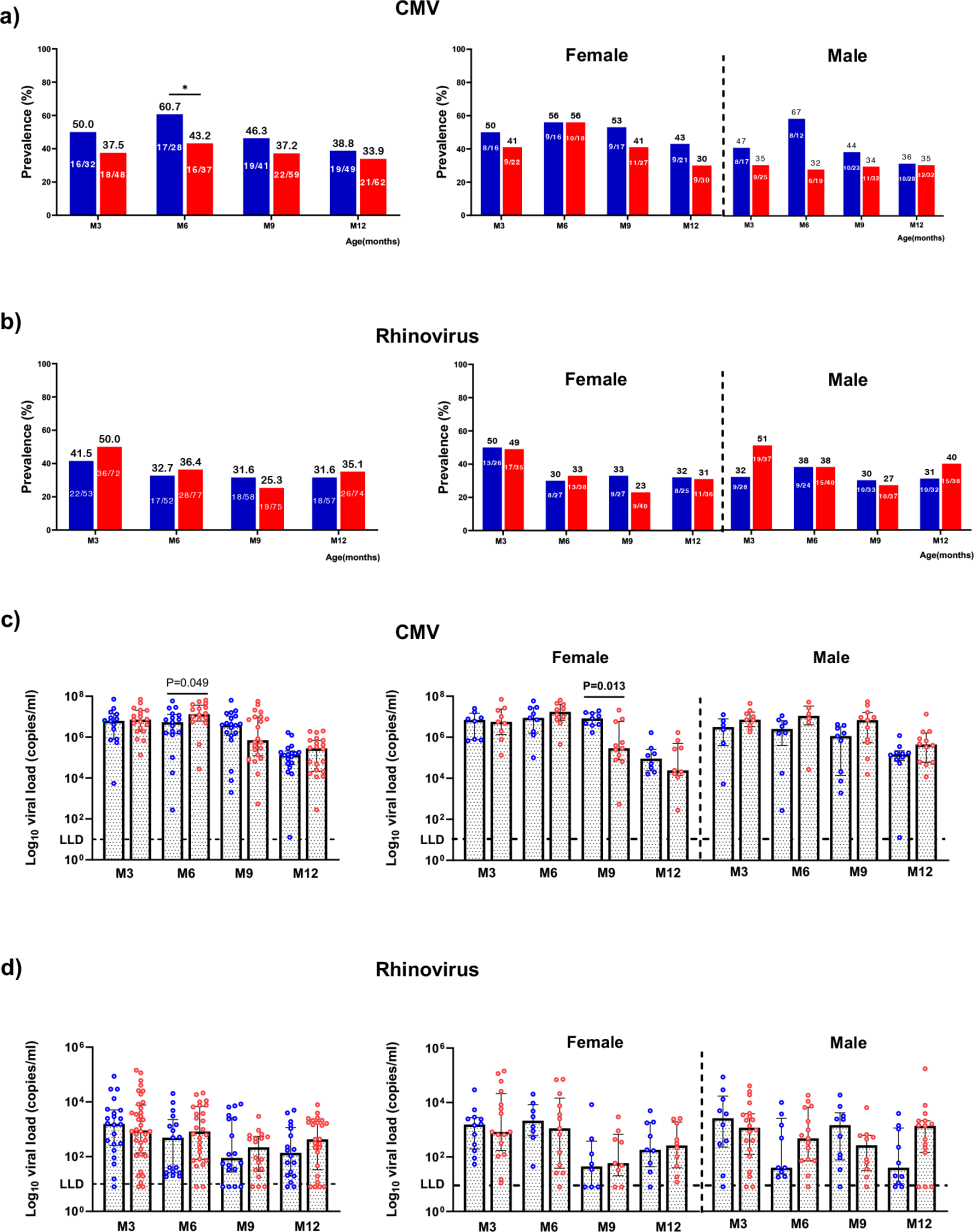
Prevalence and viremia of CMV and Rhinovirus infections in HEU (red) and HUU (blue) at 3, 6, 9 and 12 months of age. **(a)** HIV unexposed uninfected infants **(HUU)** experience a higher prevalence of CMV at 6 months of age with no sex-based differences. **(b)** Comparable prevalence of Rhinovirus infections amongst HEU and HUU infants with no sex-based differences observed. **(c)** Higher CMV viremia in HEU infants at 6 months of age. Female HUU infants experience a higher viremia at 9 months of age **d)** Comparable Rhinovirus viremia in HEU and HUU infants with no sex-based differences. Note: *Pearson’s Chi-square test was used to determine the differences in HEU and HUU infants and independent t test was used for normally distributed data while the Mann Whitney test was used to compare non-normally distributed data sets. LLD =Lower Limit of Detection.*.

To further assess the burden of infections in our study groups, the prevalence and viremia of CMV, rotavirus, malaria, rhinovirus, RSV, Influenza A & B were determined from 3 to 12 months of age in HEU and HUU infants. While infections with rotavirus, malaria, RSV, and Influenza A & B were quasi-inexistent in both groups from month 3 to 12 with a prevalence ranging from 1.4% to 5.5%, CMV and Rhinovirus infections were ubiquitous from 3 to 12 months with prevalence ranging from 33.9% to 60.7% for CMV and 25.3% to 50.0% for Rhinovirus infections by month 12 **Supplementary Figure 2A**. At month 6, CMV prevalence was higher in the HUU compared to the HEU (60.7% vs 43.2%, P = 0.03), with no other differences in prevalence amongst HEU and HUU. Sex stratified analysis of CMV, and rhinovirus infection prevalence showed no differences between male HEU and HUU, or female HEU and HUU across all timepoints **Figure 4A, B**. Sex stratified analysis of CMV and Rhinovirus viremia among PCR positives showed lower viremia in the female HEU compared to their HUU counterparts at month 9 (P = 0.013) **Figure 4C, D**. Because repeat infections might reflect weaker immune states, we also assessed the rates of new vs repeat infections to CMV and Rhinovirus. There were no differences in the rates of repeat or new infections between HEU and HUU infants **Supplementary Figure 2B**; **Supplementary Table 8**. Across the first year of life, infection burden among HEU and HUU were largely comparable with CMV with rhinovirus being the most frequent infection.

### Antibody transfer and dynamics in HEU and HUU

**Figure 5:**
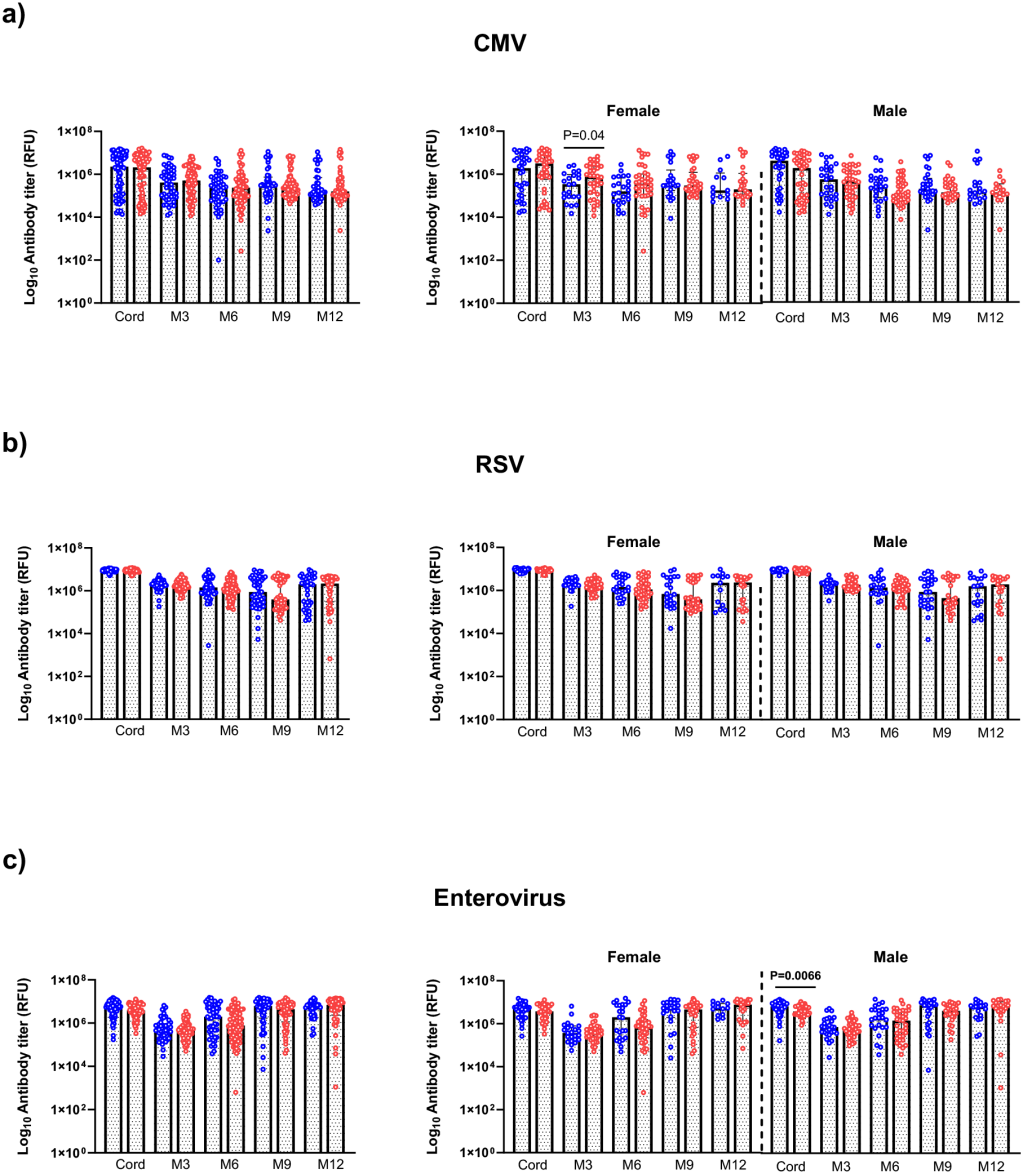
Sex stratified comparative longitudinal analysis of log_10_ transformed antibody levels to **(a)** CMV, **(b)** RSV and **(c)** Enterovirus from birth till 12 months of age in HEU (red) against their HUU (blue) counterparts. *Note: For comparative analysis, the independent t test was used for normally distributed data sets and the Mann-Whitney test for data that failed to pass normality test.*.

Following our observation of sex-effects on self-reported illness symptom patterns and PCR-based detection of common childhood infections, we evaluated humoral immune response to 3 pathogens, CMV, RSV and enterovirus to explore whether altered antibody profiles align with sex-related disparities of infection rates. Levels of antigen-specific IgG were measured at 5 time points, (birth, month 3, 6, 9 and 12). CMV, antibody levels peaked at birth and decreased with time in both groups and sexes. Female HEU had higher titers at month 3 compared to the HUU females (P = 0.04) **Figure 5A**. Maternal-infants antibody titer correlations suggest all infants lost maternal CMV antibodies by month 9 **Supplementary Figure 3A**. RSV antibody levels also peaked at birth and diminished steadily till month 9 and increased by month 12 in both groups and sexes with no difference by exposure and sex **Figure 5B**. Maternal-infant antibody level correlation showed that maternal antibodies were minimal by month 6 **Supplementary Figure 3B**. Enterovirus antibody levels were highest at birth and diminished to their lowest levels at 6 months, then started rising at months 9 with no significant differences between HEU and HUU. At birth, the male HEU infants had lower titers compared to their HUU counterparts (P = 0.0066) **Figure 5C**. Put together, these findings indicate that maternally derived protection against CMV; RSV and enterovirus declined similarly in HEU and HUU, suggesting that altered humoral transfer is unlikely to completely account for the sex specific disparities observed in infection rates.

Antibody trends described are done on independent samples. Sample sizes for each comparison= Female; cord (HUU = 37 HEU = 38), M3 (HUU = 23 HEU = 34), M6 (HUU = 24 HEU = 37), M9 (HUU = 22 HEU = 33), M12 (HUU = 13 HEU = 23) and Male; cord (HUU = 38 HEU = 40), M3 (HUU = 27 HEU = 36), M6 (HUU = 25 HEU = 36), M9 (HUU = 27 HEU = 27), M12 (HUU = 19 HEU = 21).

## Discussion

This study explored early-life growth trajectories, clinical trends associated with infection dynamics and humoral immune responses among HEU and HUU infants during their first year of life. Globally, our data suggest that HEU infants showed growth and infection profiles that was largely like that of their HUU peers, with a few transient, sex-specific differences in growth, infection dynamics and antibody responses.

The feeding practices observed in this cohort is reminiscent of what has been reported across PMTCT studies, with higher formula use amongst the HEU but similar exclusive breastfeeding rates during the first 6 months, where HIV-exposed mothers are more likely to practice formula feeding due to perceived risk of transmission (Bork et al., 2013; Wambura and Marnane, 2019). Despite similar exclusive breastfeeding rates, HEU infants exhibited sex-specific growth differences with the female HEU experiencing increased stunting at 6 months while male infants portrayed lower WAZ and HCZ at 3 months and persistent lower HAZ at 12 months. While differences in continuous Z-scores were modest and largely attenuated after adjustment, the higher prevalence of stunting suggests that even subtle shifts in growth may increase the proportion of HEU infants crossing clinically relevant thresholds. These findings are consistent with many reports from several studies showing that stunting is the most observed growth defect amongst HEU infants even when adjusted for confounders (Roux et al., 2019; Fowler et al., 2022; Neary et al., 2022). Some other studies argue that birth characteristics such as sex are predictive in growth trajectories, hence emphasizing the need to account for sex in growth analysis (Condo et al., 2015; Lane et al., 2019). Our findings show that despite the widespread use of ART, *in utero* exposure to HIV subtly affects early growth.

The temporal trends of symptoms reported by caregivers and the detection of pathogens in our study sheds light on key features of infection dynamics in infancy. The rise in prevalence of symptoms from 6 weeks to 12 months of age aligns with growing social contact, fading maternal antibodies, and increasing exposure to respiratory and enteric pathogens. Cough and rhinorrhea were the most frequent symptoms in both groups; this observation reflects the dominance of respiratory infections during infancy (Jartti et al., 2008; Wetzke et al., 2025). Although a greater proportion of HEU infants were not breastfed between 3 and 12 months, this did not translate in higher frequencies of symptom occurrence. At 9 months, HEU infants exhibited higher rhinorrhea prevalence compared to HUU, with a more pronounced pattern in males. While this may suggest a difference in susceptibility to respiratory infections at this age in the males, these observations were not consistent and should be interpreted circumspectly. The pattern was transient, with a reversal at month 12 where HEU infants showed lower frequencies of fever and cough. These findings may reflect variations in exposure, reporting or underlying biological factors; however, given that the data on symptoms are purely descriptive and rely on caregiver reporting, they should be interpreted with caution. Caregiver-reported symptoms are known to be influenced by perception and contextual factors and do not necessarily reflect underlying disease severity of immune competence (Caregivers’ perceptions of childhood… | Open Research Europe; Clinical signs that predict severe illness in children under age 2 months: a multicentre study). These observations should therefore be considered hypothesis generating rather than indicative of difference in immune competence.

During the last decades, several studies showed that HEU had increased mortality and morbidity rates when compared to HUU and this was mainly associated to respiratory infections as suggested by symptoms rates described in this study (Landes et al., 2012; Slogrove et al., 2012; Cohen et al., 2016; Slogrove et al., 2016; Lwanga et al., 2024; Infectious disease morbidity and growth among young HIV-exposed uninfected children in Jamaica, 2025). In this cohort, CMV and Rhinovirus emerged as the predominant infections, consistent with their omnipresence in early infancy (Kusel et al., 2006; Slogrove et al., 2012; Kelly et al., 2015). The comparable infection rates between HEU and HUU suggest similar exposure risks, although higher CMV viremia at 6 months in HEU might indicate temporarily reduced viral containment or delayed immune maturation. Similar exposure risks and effective viral control hypothesis are further strengthened by the quasi-existence of other infections screened in this study. This finding is consistent with studies that reported no significant difference in infection prevalence of CMV, RSV and asymptomatic malaria between HEU and HUU potentially be attributed to the effective implementation of PMTCT programs (Roux et al., 2019; Giuliano et al., 2023; Ray et al., 2024). Despite similarity in infection prevalence across groups, HEU infants remain prone to severe infection outcomes (Slogrove et al., 2012; Von Mollendorf et al., 2015; Slogrove et al., 2016; Anderson et al., 2021), underscoring the need to emphasize studies of the vulnerability of HEU on disease severity.

Patterns of humoral immune responses paralleled the infection data. Antibody levels for CMV, followed the expected maternal decay curve with no differences between groups except for momentarily higher titers in the female HEU group at 3 months of age. This suggests an efficient transfer of maternal antibodies and subsequent retention in the HEU infants with the female HEU showing a greater retention rate by month 3 compared to their HUU female peers. The enhanced retention may be influenced by immune activation and the higher susceptibility of HIV infected mothers to CMV reactivation (Barbosa et al., 2018; Mhandire et al., 2019). On the contrary, RSV and enterovirus antibody profiles showed rapid decay of antibodies by months 3 and 6 followed by a gradual recovery due to postnatal exposure. The lack of difference between HEU and HUU shows that HIV exposure did not significantly impair antibody production with the first year of life. While in this study we only quantified antigen-specific antibodies by ELISA, it did not permit us to assess B cell or cellular immune function. Complementary approaches such as B cell ELISPOT, flow cytometry-based phenotyping, and functional antibody assays (neutralization, avidity, and subclass profiling) would provide mechanistic insights into the quality and durability of immune responses and should be considered in future studies.

Put together, these findings illustrate a complex interplay between HIV exposure, growth and pathogen-specific immunity. This underscores the fact that, while maternal HIV exposure might not consistently affect infant growth or immunity, some modest and transient differences were observed, which may vary by sex. The transient nature of differences observed, supports the hypothesis that immune maturation compensates for early disparities over time.

We acknowledge several shortcomings in this study. First, the symptoms were self-reported by caregivers, this may introduce subjective bias. Secondly, despite 1014 infants timepoints from birth through months 12, our study may be underpowered for the detection of subtle changes in infection across subgroups. Finally, while antibody profiles provide a primary understanding of immune responses, cellular immune correlates would provide complementary insight.

In sum, this study offers an integrated analysis of infection dynamics, growth, and antibody responses among HEU and HUU, shedding light on comparable overall outcomes with modest transient sex-based differences, Upcoming work should integrate cellular immune phenotyping and longer-term-follow-up to elucidate the developmental and immunological trajectories of HEU.

## Data Availability

The original contributions presented in the study are included in the article/**Supplementary Material**. Further inquiries can be directed to the corresponding author.

## References

[B1] Anderson K. Kalk E. Madlala H. P. Nyemba D. C. Kassanjee R. Jacob N. . (2021). Increased infectious-cause hospitalization among infants who are HIV-exposed uninfected compared with HIV-unexposed. AIDS 35, 2327–2339. doi: 10.1097/qad.0000000000003039 34324450 PMC8563388

[B2] Barbosa N. G. Yamamoto A. Y. Duarte G. Aragon D. C. Fowler K. B. Boppana S. . (2018). Cytomegalovirus shedding in seropositive pregnant women from a high-seroprevalence population: The Brazilian Cytomegalovirus Hearing and Maternal Secondary Infection Study. Clin. Infect. Dis. 67, 743–750. doi: 10.1093/cid/ciy166 29490030 PMC6094000

[B3] Bork K. Cames C. Cournil A. Musyoka F. Ayassou K. Naidu K. . (2013). Infant feeding modes and determinants among HIV-1-infected African women in the Kesho Bora Study. J. Acquir. Immune Defic. Syndr. 62, 109–118. doi: 10.1097/qai.0b013e318277005e 23075919

[B4] Cohen C. Moyes J. Tempia S. Groome M. Walaza S. Pretorius M. . (2016). “ Epidemiology of acute lower respiratory tract infection in HIV-exposed uninfected infants,” in Pediatrics, vol. 137. (Illinois, USA: American Academy of Pediatrics). Available online at: https://publications.aap.org/pediatrics/article/137/4/e20153272/81320/Epidemiology-of-Acute-Lower-Respiratory-Tract (Accessed June 10, 2024). 10.1542/peds.2015-3272 PMC9075335 27025960

[B5] Condo J. U. Gage A. Mock N. Rice J. Greiner T. (2015). Sex differences in nutritional status of HIV-exposed children in Rwanda: a longitudinal study. Trop. Med. Int. Health 20, 17–23. doi: 10.1111/tmi.12406 25345559

[B6] Fowler M. G. Aizire J. Sikorskii A. Atuhaire P. Ogwang L. W. Mutebe A. . (2022). Growth deficits in antiretroviral and HIV-exposed uninfected versus unexposed children in Malawi and Uganda persist through 60 months of age. AIDS 36, 573–582. doi: 10.1097/qad.0000000000003122 34750297 PMC9097628

[B7] Gaensbauer J. T. Rakhola J. T. Onyango-Makumbi C. Mubiru M. Westcott J. E. Krebs N. F. . (2014). Impaired Haemophilus influenzae type b transplacental antibody transmission and declining antibody avidity through the first year of life represent potential vulnerabilities for HIV-exposed but uninfected infants. Clinical and Vaccine Immunology 21, 1661–1667. doi: 10.1128/CVI.00356-14 25298109 PMC4248779

[B8] Giuliano M. Pirillo M. F. Orlando S. Luhanga R. Mphwere R. Kavalo T. . (2023). Cytomegalovirus viremia in HIV-exposed and HIV-unexposed infants in Malawi. Acta Trop. 246, 106987. doi: 10.1016/j.actatropica.2023.106987 37454709

[B9] Hawi Ngere S. Omondi Olang’o C. Kiyuka P. Awuonda E. Sagam C. K. Ochieng B. . (2025). Caregivers’ perceptions of childhood pneumonia in Western Kenya: a theory of practice perspective. Open Research Europe 5, 221. doi: 10.12688/openreseurope.20883.1

[B10] Jartti T. Lee W.-M. Pappas T. Evans M. Lemanske R. J. Gern J. E. (2008). “ Serial viral infections in infants with recurrent respiratory illnesses,” in European respiratory journal, vol. 32. (Lausanne, Switzerland: European Respiratory Society), 314–320. Available online at: https://publications.ersnet.org/content/erj/32/2/314 (Accessed October 12, 2025). 18448489 10.1183/09031936.00161907 PMC2843696

[B11] Kelly M. S. Smieja M. Luinstra K. Wirth K. E. Goldfarb D. M. Steenhoff A. P. . (2015). “ Association of respiratory viruses with outcomes of severe childhood pneumonia in Botswana,” in PLoS one, vol. 10. (California, USA: PLoS One Journal). Available online at: https://pmc.ncbi.nlm.nih.gov/articles/PMC4431806/ (Accessed October 2, 2025). 10.1371/journal.pone.0126593 PMC4431806 25973924

[B12] Kusel M. M. H. Klerk N. Holt P. G. Kebadze T. Johnston S. L. Sly P. D. (2006). Role of respiratory viruses in acute upper and lower respiratory tract illness in the first year of life: a birth cohort study. Pediatr. Infect. Dis. J. 25, 680–686. doi: 10.1097/01.inf.0000226912.88900.a3 16874165

[B13] Landes M. Lettow M. Chan A. K. Mayuni I. Schouten E. J. Bedell R. A. (2012). Mortality and health outcomes of HIV-exposed and unexposed children in a PMTCT cohort in Malawi. PloS One 7, e47337. doi: 10.1371/journal.pone.0047337 23082157 PMC3474798

[B14] Lane C. E. Bobrow E. A. Ndatimana D. Ndayisaba G. F. Adair L. S. (2019). “ Determinants of growth in HIV-exposed and HIV-uninfected infants in the Kabeho Study,” in Matern child nutr. (Bethesda, USA: National Library of Medicine), vol. 15. 10.1111/mcn.12776 PMC6667835 30609287

[B15] Lwanga C. Aber P. Tickell K. D. Ngari M. M. Mukisa J. Atuhairwe M. . (2024). “ Impact of HIV exposure without infection on hospital course and mortality among young children in sub-Saharan Africa: a multi-site cohort study,” in BMC medicine, vol. 22 (Berlin Germany: Springer Nature), 573. doi: 10.1186/s12916-024-03790-5 39627711 PMC11613948

[B16] Mhandire D. Duri K. Kaba M. Mhandire K. Musarurwa C. Chimusa E. . (2019). “ Seroprevalence of cytomegalovirus infection among HIV-infected and HIV-uninfected pregnant women attending antenatal clinic in Harare, Zimbabwe,” in Viral immunol, vol. 32. (Bethesda, USA: National Library of Medicine), 289–295. 31347990 10.1089/vim.2019.0024 PMC6751388

[B17] Neary J. Langat A. Singa B. Kinuthia J. Itindi J. Nyaboe E. . (2022). “ Higher prevalence of stunting and poor growth outcomes in HIV-exposed uninfected than HIV-unexposed infants in Kenya,” in AIDS, vol. 36. (Pennsilvania, USA: Wolters Kluwer Health), 605. Available online at: https://journals.lww.com/aidsonline/abstract/2022/03150/higher_prevalence_of_stunting_and_poor_growth.14.aspx (Accessed June 6, 2024). 34750290 10.1097/QAD.0000000000003124 PMC8985586

[B18] Nlend A. E. N. Avenec P. Ngoué J. E. Sandie A. B. (2023). Morbidity and mortality of HIV-exposed uninfected infants in a tertiary referral facility in Yaoundé, Cameroon. Int. J. MCH AIDS 12, e663. doi: 10.21106/ijma.663 38312496 PMC10753404

[B19] Patel S. M. Jallow S. Boiditswe S. Madhi S. A. Feemster K. A. Steenhoff A. P. . (2019). “ Placental transfer of respiratory syncytial virus antibody among HIV-exposed, uninfected infants,” in J pediatric infect dis soc, vol. 9. (Oxford, UK: Oxford University Press), 349–356. Available online at: https://pmc.ncbi.nlm.nih.gov/articles/PMC7358043/ (Accessed November 20, 2025). 10.1093/jpids/piz056 PMC7358043 31549157

[B20] Pierre R. B. Fulford T.-A. Lewis K. Palmer P. Walters C. Christie C. D. C. (2016). Infectious disease morbidity and growth among young HIV-exposed uninfected children in Jamaica. Revista Panamericana de Salud Pública 40, 401–409. Available online at: https://www.scielosp.org/article/rpsp/2016.v40n6/401-409/ (Accessed October 2, 2025). 28718488

[B21] Ray J. E. Dobbs K. R. Ogolla S. O. Daud I. I. Midem D. Omenda M. M. . (2019). “ Reduced transplacental transfer of antimalarial antibodies in Kenyan HIV-exposed uninfected infants,” in Open forum infect dis, vol. 6. (Oxford, UK: Oxford University Press). Available online at: https://pmc.ncbi.nlm.nih.gov/articles/PMC6563943/ (Accessed November 20, 2025). 10.1093/ofid/ofz237 PMC6563943 31214627

[B22] Ray J. E. Dobbs K. R. Ogolla S. O. Daud I. I. Vulule J. Sumba P. O. . (2024). “ Clinical and immunological outcomes of HIV-exposed uninfected and HIV-unexposed uninfected children in the first 24 months of life in Western Kenya,” in BMC infect dis. (Berlin, Germany: Springer Nature), vol. 24, 156. Available online at: https://bmcinfectdis.biomedcentral.com/articles/10.1186/s12879-024-09051-3 (Accessed March 18, 2024). 38302888 10.1186/s12879-024-09051-3 PMC10835872

[B23] Roux S. Abrams E. J. Donald K. A. Brittain K. Phillips T. K. Nguyen K. K. . (2019). Growth trajectories of breastfed HIV-exposed uninfected and HIV-unexposed children under conditions of universal maternal antiretroviral therapy: a prospective study. Lancet Child Adolesc. Health 3, 234–244. doi: 10.1016/s2352-4642(19)30007-0 30773459

[B24] Salvi M. Fioretti B. Alberti M. Scarvaglieri I. Arsuffi S. Tiecco G. . (2025). “ Understanding HIV-exposed uninfected children: A narrative review,” in Viruses, vol. 17. (Basel, Switzerland: publisher). Available online at: https://www.mdpi.com/1999-4915/17/3/442 (Accessed November 19, 2025). 10.3390/v17030442 PMC11945683 40143369

[B25] Semmes E. C. Chen J.-L. Goswami R. Burt T. D. Permar S. R. Fouda G. G. (2021). “ Understanding early-life adaptive immunity to guide interventions for pediatric health,” in Front immunol, vol. 11. (Lausanne, Switzerland: Frontiers). Available online at: https://www.frontiersin.org/journals/immunology/articles/10.3389/fimmu.2020.595297/full (Accessed October 2, 2025). 10.3389/fimmu.2020.595297 PMC7858666 33552052

[B26] Slogrove A. L. Goetghebuer T. Cotton M. F. Singer J. Bettinger J. A. (2016). “ Pattern of infectious morbidity in HIV-exposed uninfected infants and children,” in Front immunol, vol. 7. (Oxford, UK: Oxford University Press). Available online at: http://journal.frontiersin.org/Article/10.3389/fimmu.2016.00164/abstract (Accessed October 2, 2025). 10.3389/fimmu.2016.00164 PMC4858536 27199989

[B27] Slogrove A. Reikie B. Naidoo S. De Beer C. Ho K. Cotton M. . (2012). “ HIV-exposed uninfected infants are at increased risk for severe infections in the first year of life,” in J trop pediatr, vol. 58. (Lausanne, Switzerland: Frontiers), 505–508. Available online at: https://pmc.ncbi.nlm.nih.gov/articles/PMC3612013/ (Accessed June 6, 2024). 22555385 10.1093/tropej/fms019 PMC3612013

[B28] Steeg L. Klein S. L. (2016). “ SeXX matters in infectious disease pathogenesis,” in PLOS pathogens, vol. 12. (California, USA: Public Library of Science). Available online at: https://journals.plos.org/plospathogens/article?id=10.1371/journal.ppat.1005374 (Accessed October 3, 2025). 10.1371/journal.ppat.1005374 PMC4759457 26891052

[B29] Sudfeld C. R. Lei Q. Chinyanga Y. Tumbare E. Khan N. Dapaah-Siakwan F. . (2016). “ Linear growth faltering among HIV-exposed uninfected children,” in J acquir immune defic syndr, vol. 73. (Bethesda, USA: National Library of Medicine), 182–189. Available online at: https://pmc.ncbi.nlm.nih.gov/articles/PMC5023451/ (Accessed October 2, 2025). 27116046 10.1097/QAI.0000000000001034 PMC5023451

[B30] The Young Infants Clinical Signs Study Group . (2008). Clinical signs that predict severe illness in children under age 2 months: a multicentre study. The Lancet 371, 135–142. doi: 10.1016/S0140-6736(08)60106-3 18191685

[B31] Tiwari R. Singa B. O. Lihanda P. Diakhate M. M. Ochola E. Bunyige L. . (2025). “ Differences in growth trajectories in breastfed HIV-exposed uninfected and HIV-unexposed infants in Kenya: An observational cohort study,” in PLOS medicine, vol. 22. (California, USA: Public Library of Science). Available online at: https://journals.plos.org/plosmedicine/article?id=10.1371/journal.pmed.1004781 (Accessed November 19, 2025). 10.1371/journal.pmed.1004781 PMC12578329 41144576

[B32] Tshiambara P. Hoffman M. Legodi H. Botha T. Mulol H. Pisa P. . (2023). Comparison of feeding practices and growth of urbanized African infants aged 6–12 months old by maternal HIV status in Gauteng Province, South Africa. Nutrients 15, 1500. doi: 10.3390/nu15061500 36986230 PMC10053312

[B33] UNAIDS . (2023). The path that ends AIDS: UNAIDS Global AIDS Update 2023. UNAIDS. Available online at: https://www.unaids.org/en/resources/documents/2023/global-aids-update-2023 (Accessed December 12, 2025).

[B34] von Mollendorf C. von Gottberg A. Tempia S. Meiring S. de Gouveia L. Quan V. . (2015). “ Increased risk for and mortality from invasive pneumococcal disease in HIV-exposed but uninfected infants aged <1 year in South Africa, 2009–2013,” in Clinical infectious diseases, vol. 60. (Oxford, United Kingdom: Oxford University Press), 1346–1356. Available online at: https://academic.oup.com/cid/article-lookup/doi/10.1093/cid/civ059 (Accessed June 6, 2024). 25645212 10.1093/cid/civ059

[B35] Wambura J. N. Marnane B. (2019). “ Undernutrition of HEU infants in their first 1000 days of life: A case in the urban-low resource setting of Mukuru Slum, Nairobi, Kenya,” in Heliyon, vol. 5. (Oxford, England: Elsevier). Available online at: https://www.cell.com/heliyon/abstract/S2405-8440(19)35733-0 (Accessed October 20, 2025). 10.1016/j.heliyon.2019.e02073 PMC6658733 31372539

[B36] Wedderburn C. J. Bondar J. Lake M. T. Nhapi R. Barnett W. Nicol M. P. . (2024). “ Risk and rates of hospitalisation in young children: A prospective study of a South African birth cohort,” in PLOS global public health, vol. 4. (California, USA: Public Library of Science). Available online at: https://journals.plos.org/globalpublichealth/article?id=10.1371/journal.pgph.0002754 (Accessed November 19, 2025). 10.1371/journal.pgph.0002754 PMC10793893 38232126

[B37] Wetzke M. Lange M. Koerner-Rettberg C. Kiefer A. Kabesch M. Armbrust S. . (2025). “ RSV is the main cause of severe respiratory infections in infants and young children in Germany - data from the prospective, multicenter PAPI study 2021–2023,” in Infection, vol. 53. (Berlin, Germany: Springer Nature), 1715–1723. doi: 10.1007/s15010-025-02484-1. 39971837 PMC12460574

