# Supplementary material for: Growth, infection, and humoral immunity in children who are HIV exposed and uninfected
Source: Front Cell Infect Microbiol. 2026 Jul 9;16:1816838. doi: 10.3389/fcimb.2026.1816838 (PMC13391899; doi:10.3389/fcimb.2026.1816838)
Supplement: Supplementary file 1 [file SupplementaryFile1.docx]

**Supplementary Materials**

**Supplementary table 1:** Primer and probe sequences used for multiplex RT-PCR detection of pathogens

| **Pathogen** | **Target** | **Forward primer** | **Reverse primer** | **Probe** |
| --- | --- | --- | --- | --- |
| CMV | E1 | GAGCAGACTCTCAGAGGATCGG | CTTGGTTATCAGAGGCCGCTT | FAM-CATGCAGATCTCCTCAATGCGGCG-TQ2 |
| Albumin | Albumin gene | GTGAACAGGCGACCATGCT | TGAAACATTCACCTTCCATGC | HEX-TCAGCTCTGGAAGTCGATGAAACATACGTTC-TQ2 |
| RSV | N gene | GCAAATATGGAAACATACGTGAACA | TGCATCACTTACAATATGGGTGC | CTTCACGAAGGCTCCACATACACAGCWG 5’-FAM, 3’- TQ2 |
| Rhinovirus | 5’ UTR | GGTGTGAAGACTCGCATGTGCT | GGACCGACTACTTTGGGTGTCC | CCGGCCCCTGAATGYGGCTAAYC 5’-CY5, 3’-MGB |
| Influenza A | M gene | GACCRATCCTGTCACCTCTGAC | TAGACGATTTGTCCAAAATGCCCT | TGCAGTCCTCGCTCACTGGGCACG 5’-HEX, 3’-TQ2 |
| Influenza B | HA gene | AAATACGGTGGATTAAATAAAAGCAA | TTTCTTCGGAGCTATTGCTGG | CACCCATATTGGGCAATTTCCTAT 5’-TEXAS RED, 3’-BHQ2 |
| Rotavirus | NSP3 gene | ACCATCTACACATGACCCTC | GGGGCGTTATGTGACC | FAM ATGAGCACAATAGTTAAAAGCTAACACTGTCAA TQ2 |
| *Plasmodium* spp. | 18s | TATTGCTTTTGAGAGGTTTTGTTACTTTG | ACCTCTGACATCTGAATACGAATGC | FAM-ACGGGTAGTCATGATTGAGTT-TQ2 |

**Supplementary table 2:** Cycling conditions

| **Pathogen panel** | **Reverse transcription** | **Initial denaturation** | **Cycling steps**  **(Den, Ann, Elon)** | **Total cycles** |
| --- | --- | --- | --- | --- |
| Multiplex DNA (CMV + Albumin) | / | 95°C 2min | 94°C 40s, 57°C 30s, 72°C 1min | 41 |
| Multiplex RNA (RSV, Rhino, Influenza A & B) | 55 °C 10min | 95 °C 5min | 94°C 15s, 60°C 20s, 72°C 40s | 42 |
| Rotavirus (singleplex) | 55 °C 10min | 95 °C 5min | 94°C 10s, 60°C 20s, 60°C 40s | 40 |
| Malaria (singleplex) | / | 95°C 10min | 95°C 15s, 58°C 20s, 58°C 40s | 47 |

**Supplementary Table 3:** Sex stratified Z-score indices of infants at 3, 6, 9 and 12 months of age by HIV exposure status.

| Female | | | | | | | | | | | | | |
| --- | --- | --- | --- | --- | --- | --- | --- | --- | --- | --- | --- | --- | --- |
| Age (Months) | **Month 3** | | | **Month 6** | | | **Month 9** | | | **Month 12** | | | |
| Growth parameter | **HUU (N= 31)** | **HEU (N=39)** | **P value** | **HUU (N=25)** | **HEU (N=43)** | **P value** | **HUU (N=26)** | **HEU (N=39)** | **P value** | **HUU (N=25)** | **HEU (N=37)** | **P value** |  |
| WHZ mean (SD) | 0.13  (±1.33) | 0.25  (±1.38) | 0.71 ^b^ | 0.80 (±1.29) | 1.00 (±2.03) | 0.93 ^a^ | 0.61 (±1.17) | 0.47 (±1.38) | 0.67 ^b^ | 0.69 (4.54) | 0.54 (±2.64) | 0.57 ^a^ |  |
| HAZ mean (SD) | 0.61 (± 1.01) | 0.58  (± 1.20) | 0.93 ^b^ | 0.31 (±1.11) | -0.33 (±1.51) | 0.07 ^b^ | -0.27 (±1.37) | -0.10 (±1.38) | 0.30 ^a^ | -1.28 (±2.47) | -0.17 (±2.80) | 0.32 ^a^ |  |
| WAZ mean (SD) | 0.45 (±1.08) | 0.46 (±1.13) | 0.97 ^b^ | 0.71 (±1.12) | 0.43 (±1.29) | 0.39 ^b^ | 0.25 (±1.24) | 0.19 (±1.00) | 0.83 ^b^ | 0.42 (±2.23) | 0.45 (±3.02) | 0.05 ^a^ |  |
| BAZ mean (SD) | 0.15 (±1.31) | 0.26 (±1.39) | 0.75 ^b^ | 0.73 (±1.37) | 0.88 (±2.05) | 0.99 ^a^ | 0.56 (±1.18) | 0.41 (±1.44) | 0.78 ^a^ | 0.58 (±3.93) | 0.67 (±2.77) | 0.60 ^a^ |  |
| HCZ mean (SD) | 1.08 (±1.42) | 0.76 (±1.66) | 0.40 ^b^ | 0.71 (±1.20) | 0.53 (±1.55) | 0.62 ^a^ | 0.58 (±0.99) | 0.76 (±1.44) | 0.63 ^a^ | 0.69 (±1.34) | 1.24 (±0.35) | 0.55 ^a^ |  |
| Male | | | | | | | | | | | | |  |
| Age (Months) | **Month 3** | | | **Month 6** | | | **Month 9** | | | **Month 12** | | |  |
| Growth parameter | **HUU (N= 31)** | **HEU (N=39)** | **P value** | **HUU (N=29)** | **HEU (N=40)** | **P value** | **HUU (N=32)** | **HEU (N=39)** | **P value** | **HUU (N=32)** | **HEU (N=36)** | **P value** |  |
| WHZ mean (SD) | 0.72 (±1.06) | 0.57 (±1.80) | 0.69 ^b^ | 0.34 (±2.93) | 0.19 (1.40) | 0.16 ^a^ | -0.19 (±2.71) | -0.10 (±2.60) | 0.97 ^a^ | -0.16 (±2.90) | 0.72 (±1.41) | 0.36 ^a^ |  |
| HAZ mean (SD) | 0.68 (±2.06) | -0.38 (±1.82) | 0.09 ^a^ | -0.41 (±2.31) | -0.11 (±1.80) | 0.80 ^a^ | -0.34 (±1.40) | -0.77 (±1.55) | 0.52 ^a^ | -0.12 (±2.73) | -1.10 (±1.79) | 0.13 ^a^ |  |
| WAZ mean (SD) | 1.07 (±1.94) | 0.05 (±1.35) | **0.04 ^a^** | 0.11 (±2.24) | -0.04 (±1.21) | 0.43 ^a^ | -0.37 (±2.15) | -0.57 (±1.82) | 0.49 ^a^ | -0.12 (±2.99) | -0.02 (±1.05) | 0.95 ^a^ |  |
| BAZ mean (SD) | 0.88 (±1.31) | 0.39 (±1.61) | 0.17 ^b^ | 0.25 (±2.89) | 0.04 (±1.41) | 0.14 ^a^ | -0.24 (±2.74) | 0.12 (±2.64) | 0.98 ^a^ | -0.07 (±3.12) | 0.84 (±1.57) | 0.20 ^a^ |  |
| HCZ mean (SD) | 1.39 (±2.38) | 0.43 (±1.17) | **0.03 ^b^** | 0.42 (±1.40) | 0.55 (±1.48) | 0.72 ^b^ | 0.50 (±0.26) | 0.30 (±1.59) | 0.59 ^b^ | 1.00 (±2.25) | 0.36 (±1.33) | 0.39 ^a^ |  |

*Note: The independent t test was used for normally distributed data while the Mann Whitney test was used to compare non-normally distributed data sets. Z-scores indices at ages 3, 6, 9 and 12 months were computed using WHO Anthro* *software of 2010 v3.2.2.*

**WHZ=** Weight-for-Height; **HAZ=** Height-for-Age; **WAZ=** Weight-for-Age; **BAZ=** BMI-for-Age; **HCZ=** Head Circumference; **^a^ =** Mann-Whitney test**; *^b^* =** Independent t test.

**Supplementary table 4**: Sex stratified nutritional classification of infants at 3,6,9 and 12 months.

| Female | | | | | | | | | | | | |
| --- | --- | --- | --- | --- | --- | --- | --- | --- | --- | --- | --- | --- |
| Age (Months) | **Month 3** | | | **Month6** | | | **Month 9** | | | **Month 12** | | |
| Nutritional classification | **HUU (N= 30)** | **HEU (N=39)** | **P value** | **HUU (N=26)** | **HEU (N=42)** | **P value** | **HUU (N=26)** | **HEU (N=39)** | **P value** | **HUU (N=25)** | **HEU (N=36)** | **P value** |
| Wasting, n (%) ^c^ | 1(3.3) | 2(5.1) | >0.99 | 0(0) | 1(2.3) | >0.99 | 0(0) | 2(5.1) | 0.51 | 2(8) | 1(2.8) | 0.56 |
| Stunting, n (%) ^c^ | 1(3.3) | 1(2.6) | >0.99 | 0(0) | 9(21.4) | **0.01** | 2(7.7) | 2(5.1) | >0.99 | 8(32) | 6(16.7) | 0.22 |
| Underweight, n (%) ^c^ | 2(6.7) | 0(0) | 0.19 | 0(0) | 1(2.3) | >0.99 | 1(3.7) | 0(0) | 0.41 | 2(8) | 5(13.9) | 0.69 |
| Microcephaly, n (%) ^c^ | 1(3.3) | 2(5.1) | >0.99 | 0(0) | 1(2.3) | >0.99 | 0(0) | 1(2.6) | >0.99 | 1(4) | 0(0) | 0.41 |
| Male | | | | | | | | | | | | |
| Age (Months) | **Month 3** | | | **Month6** | | | **Month 9** | | | **Month 12** | | |
| Nutritional classification | **HUU (N= 31)** | **HEU (N=39)** | **P value** | **HUU (N=29)** | **HEU (N=40)** | **P value** | **HUU (N=32)** | **HEU (N=39)** | **P value** | **HUU (N=32)** | **HEU (N=36)** | **P value** |
| Wasting, n (%) ^c^ | 0(0) | 1(2.6) | >0.99 | 2(7.1) | 2(5) | >0.99 | 2(6.3) | 4(103) | 0.68 | 4(12.5) | 2(8.3) | 0.69 |
| Stunting, n (%) ^c^ | 0(0) | 7(18.4) | **0.01** | 6(21.4) | 8(20.0) | >0.99 | 3(9.4) | 9(23.1) | 0.20 | 4(12.5) | 14(38.9) | **0.03** |
| Underweight, n (%) ^c^ | 1(3.2) | 2(5.1) | 0.62 | 1(3.6) | 2(5) | >0.99 | 3(9.4) | 2(5.1) | 0.65 | 4(12.5) | 1(2.9) | 0.18 |
| Microcephaly, n (%) ^c^ | 1(3.2) | 1(2.6) | >0.99 | 1(3.6) | 3(8.3) | 0.63 | 1(3.1) | 2(5.1) | >0.99 | 0(0) | 1(2.8) | >0.99 |

***Note:*** *Pearson's Chi-square/Fisher's exact test was used for categorical data to determine the differences in HEU and HUU infants.*

**Wasting** = WHZ < -2, **Stunting** = HAZ < -2, **Underweight** =WAZ < -2, **Microcephaly** =HCZ < -2, **^c^** **=** Chi Square test

**Supplementary Table 5:** Mixed-effects regression models of growth outcomes in HEU versus HUU infants

| **Females (HUU n=30; HEU n=39)** | | | | |
| --- | --- | --- | --- | --- |
| **Outcome** | **Unadjusted β (95% CI)** | **P value** | **Adjusted β* (95% CI)** | **P value** |
| WAZ | –0.76 (–1.64, 0.13) | 0.094 | 0.07 (–0.68, 0.83) | 0.848 |
| HAZ | –0.96 (–2.10, 0.19) | 0.102 | –0.03 (–0.69, 0.63) | 0.932 |
| WHZ | –0.28 (–1.65, 1.08) | 0.686 | 0.07 (–0.61, 0.75) | 0.841 |
| BAZ | –0.33 (–1.74, 1.07) | 0.640 | 0.06 (–0.64, 0.76) | 0.864 |
| HCZ | –0.57 (–1.62, 0.48) | 0.285 | –0.08 (–0.76, 0.61) | 0.825 |
| MUACZ | 0.32 (–0.40, 1.04) | 0.385 | –0.10 (–0.62, 0.43) | 0.720 |
| **Males (HUU n=31; HEU n=39)** | | | | |
| **Outcome** | **Unadjusted β (95% CI)** | **P value** | **Adjusted β* (95% CI)** | **P value** |
| WAZ | –0.46 (–1.29, 0.37) | 0.279 | 0.21 (–0.45, 0.88) | 0.533 |
| HAZ | –0.73 (–1.44, –0.02) | 0.043 | –0.28 (–1.12, 0.56) | 0.516 |
| WHZ | –0.25 (–1.05, 0.56) | 0.545 | 0.69 (–0.18, 1.57) | 0.122 |
| BAZ | –0.23 (–1.05, 0.58) | 0.579 | 0.65 (–0.25, 1.54) | 0.155 |
| HCZ | –0.39 (–1.04, 0.25) | 0.233 | –0.34 (–1.07, 0.38) | 0.356 |
| MUACZ | –0.27 (–0.89, 0.35) | 0.396 | 0.08 (–0.45, 0.60) | 0.779 |

*Adjusted for age, feeding mode, Socioeconomic status; Maternal and gestational age. β indicates the mean change in Z score between HEU and HUU infants.

**Supplementary Table 6:** Sensitivity analysis excluding formula-fed infants

| **Females** | | |
| --- | --- | --- |
| **Outcome** | **Adjusted β (95% CI)** | **P value** |
| WAZ | –0.76 (–2.00, 0.47) | 0.225 |
| HAZ | –0.59 (–1.70, 0.52) | 0.299 |
| WHZ | –0.17 (–1.77, 1.43) | 0.835 |
| BAZ | –0.49 (–2.13, 1.15) | 0.558 |
| HCZ | –1.10 (–2.11, –0.10) | 0.031 |
| MUACZ | 0.01 (–0.70, 0.73) | 0.970 |
| **Males** | | |
| **Outcome** | **Adjusted β (95% CI)** | **P value** |
| WAZ | –0.65 (–1.81, 0.51) | 0.270 |
| HAZ | –0.82 (–1.86, 0.22) | 0.123 |
| WHZ | –0.08 (–1.42, 1.26) | 0.909 |
| BAZ | –0.15 (–1.51, 1.22) | 0.834 |
| HCZ | –0.66 (–1.69, 0.36) | 0.203 |
| MUACZ | 0.04 (–0.70, 0.79) | 0.912 |

β indicates the mean change in Z score between HEU and HUU infants.

**Supplementary Table 7:** Association between ART duration and growth outcomes among HEU infants

| **Females** | | |
| --- | --- | --- |
| **Outcome** | **ART β (95% CI)** | **P value** |
| WAZ | –0.0016 (–0.0094, 0.0062) | 0.688 |
| HAZ | –0.0044 (–0.0115, 0.0027) | 0.228 |
| WHZ | 0.0014 (–0.0051, 0.0080) | 0.666 |
| BAZ | 0.0017 (–0.0051, 0.0086) | 0.618 |
| HCZ | 0.0035 (–0.0041, 0.0112) | 0.365 |
| MUACZ | 0.0003 (–0.0053, 0.0059) | 0.920 |
| **Males** | | |
| **Outcome** | **ART β (95% CI)** | **P value** |
| WAZ | 0.0028 (–0.0017, 0.0074) | 0.224 |
| HAZ | 0.0004 (–0.0067, 0.0076) | 0.910 |
| WHZ | 0.0031 (–0.0039, 0.0101) | 0.389 |
| BAZ | 0.0032 (–0.0038, 0.0102) | 0.367 |
| HCZ | 0.0010 (–0.0046, 0.0066) | 0.728 |
| MUACZ | –0.0004 (–0.0040, 0.0033) | 0.846 |

β indicates the mean change in Z score between HEU and HUU infants.

**Supplementary figure 1:** Distribution of infant feeding modes over time in HEU and HUU infants.
Stacked bars represent the percentage of infants in each feeding category at months 3, 6, 9, and 12, stratified by HIV exposure status. Feeding categories include non-breastfeeding (NBF), mixed feeding (MF), and breastfeeding dominant (BFD). Corresponding frequencies and P values are shown in the table below.

***Note:*** *P values were calculated using the Fishers exact test. Breastfeeding dominant group comprise of those taking exclusively breastmilk and does taking breastmilk and solid food at later timepoints. Percentages at each visit were calculated using infants with available data at the respective time point.*

**Supplementary figure 2:** a) Prevalence of 7 common childhood infections by HIV exposure status from month 3 to 12. b) New vs repeat proportion trend for CMV and Rhinovirus infections from month 3 to 12.

Note: *Pearson's Chi-square test was used to determine the differences in HEU and HUU infants.* *New infections represent first detection at a given visit; repeat infections represent detections following a prior positive result.*

**Supplementary Table 8:** Statistics of New and Repeat-positive CMV and Rhinovirus detections by HIV exposure status

| **CMV** | | | | | **Rhinovirus** | | | |
| --- | --- | --- | --- | --- | --- | --- | --- | --- |
| Timepoint | Outcome | HUU n/N (%) | HEU n/N (%) | P-value | Outcome | HUU n/N (%) | HEU n/N (%) | P-value |
| Month 3 | New | 16/33 (48.5%) | 18/48 (37.5%) | 0.325 | New | 22/54 (40.7%) | 36/72 (50.0%) | 0.302 |
|  | Repeat | NA | NA |  | Repeat | NA | NA |  |
| Month 6 | New | 16/28 (57.1%) | 14/37 (37.8%) | 0.122 | New | 11/51 (21.6%) | 18/79 (22.8%) | 0.871 |
|  | Repeat | 1/5 (20.0%) | 2/8 (25.0%) | 0.835 | Repeat | 6/40 (15.0%) | 10/64 (15.6%) | 0.932 |
| Month 9 | New | 9/41 (22.0%) | 7/59 (11.9%) | 0.176 | New | 7/59 (11.9%) | 8/77 (10.4%) | 0.786 |
|  | Repeat | 10/41 (24.4%) | 15/59 (25.4%) | 0.907 | Repeat | 11/59 (18.6%) | 11/77 (14.3%) | 0.494 |
| Month 12 | New | 6/49 (12.2%) | 3/62 (4.8%) | 0.156 | New | 7/57 (12.3%) | 5/74 (6.8%) | 0.277 |
|  | Repeat | 13/49 (26.5%) | 18/62 (29.0%) | 0.771 | Repeat | 11/57 (19.3%) | 21/74 (28.4%) | 0.230 |

*Note: P values were calculated using the Fishers exact test. Repeat-positive detections may reflect persistent viral shedding or reinfection and cannot be distinguished by PCR alone.*

**Supplementary figure 3:** Correlation of maternal and cord antibodies to months 3, 6, 9 and 12 titers in HEU and HUU infants.

*Note: Only correlations with p < 0.05 appear on correlograms. The size of the circle represents the correlation coefficient, with positive and negative values in blue and red shades respectively.*
